# Taking action early to manage emergence of acquired resistance to osimertinib or other third generation EGFR inhibitors

**DOI:** 10.18632/oncoscience.544

**Published:** 2021-09-15

**Authors:** Guangzhi Ma, Shi-Yong Sun

**Keywords:** lung cancer, EGFR-TKIs, osimertinib, acquired resistance

Lung cancer, which consists of over 80% non-small cell lung cancer (NSCLC), remains the leading cause of cancer mortality globally, accounting for approximately 180 million deaths in 2020 [1]. The first-generation epidermal growth factor receptor (EGFR) tyrosine kinase inhibitors (EGFR-TKIs) such as gefitinib and erlotinib have proven effective in advanced lung cancers harboring EGFR mutations. Although they show remarkable efficacies, drug-resistance to first-generation EGFR-TKIs is inevitable within 1 year due to the emergence of acquired EGFR mutations such as T790M [2]. Accordingly, the third-generation EGFR-TKI, osimertinib (AZD9291), which functions as a mutation-selective EGFR-TKI by targeting both T790M and other common EGFR activating mutations including L858R and 19Del, was developed [3]. Apart from being a second-line remedy to first-generation TKI resistance, osimertinib has now become a standard first-line treatment for advanced NSCLC due to its efficacy based on overall survival (OS) and progression-free survival (PFS) [4, 5]. Nonetheless, drug resistance to osimertinib still occurs whether it is used as a first-line or second-line treatment within 19 and 10 months, respectively [6]. Therefore, to improve patient survival duration, having a better understanding of osimertinib resistance and developing strategies to prevent drug-resistance are of vital importance.

Aberrant MEK/ERK pathway signaling is prevalent among various malignancies including NSCLC. Thus, several inhibitors which target the MEK/ERK signaling pathway, such as trametinib and ulixertinib, were developed [7]. It has been demonstrated that effective suppression of the MEK/ERK signaling pathway is a critical mechanism for third-generation EGFR-TKIs, such as osimertinib, to exert their therapeutic efficacies against EGFR mutant NSCLC cells [8, 9]. Moreover, the aberrant activation of Ras/Raf/MEK/ERK signaling pathway has been connected to the development of acquired resistance to third generation EGFR-TKIs [9]. Accordingly, targeting MEK/ERK signaling with either MEK or ERK inhibitors could effectively overcome acquired resistance to osimertinib of EGFR-mutant NSCLC cells and tumors as demonstrated by us and others [8–10]. While this strategy is potentially effective and promising in overcoming acquired resistance to osimertinib and other third-generation EGFR-TKIs, it is still a passive action after cancer cells have developed resistance to the treatment. Is there an effective way to stop or delay the development of acquired resistance to osimertinib and other third-generation EGFR-TKIs?

Results from our recent study have demonstrated the effectiveness of targeting MEK/ERK in delaying or abrogating emergence of acquired resistance to osimertinib in preclinical settings [11]. In this study, we first found that osimertinib exerted a transient suppressive effect on MEK/ERK signaling, supporting the rationale for inclusion of a MEK or ERK inhibitor to achieve continuous or long-term MEK/ERK inhibition. As a result, synergistic effects that decrease cell survival of EGFR-mutant NSCLC cell lines were detected when osimertinib was combined with a MEK or ERK inhibitor. The combination treatment also resulted in the highest level of apoptosis compared to each single agent treatment, yet such phenomenon was not observed in NSCLC cell lines with wild-type EGFR. Very importantly, targeting MEK/ERK in combination with osimertinib could effectively decrease the survival of several cell clones that are intrinsically resistant to osimertinib with enhanced induction of apoptosis. In our *in vitro* models, osimertinib resistant clones were detected after 39 days of osimertinib single agent treatment, whereas no visible resistant clones were observed over 100 days in the group treated with osimertinib and trametinib combination either in a concurrent or intermittent way. These findings were further validated in our *in vivo* study conducted with mice carrying xenografts from osimertinib sensitive NSCLC cells. Acquired resistance was observed after 30 days of osimertinib treatment as a single agent, whereas the combination of osimertinib and trametinib continued to suppress tumor growth over a treatment period of 3 months. Moreover, the outcomes of the combination treatments were achieved whether trametinib was administered concurrently or intermittently with osimertinib. The body weight of the mice in the combination group was not significantly decreased compared to other groups, indicating a safe and well-tolerated approach. One point worth mentioning is that some mice had no detectable tumors in combination treatment groups; this collectively accounted for a cure rate of 27.8% (5 of 18 mice in total), suggesting that some mice may achieve long-term remission with these combination treatments. This outcome clearly has highly meaningful potential in the clinic.

Our study set out to determine a potential strategy to prevent or delay emergence of osimertinib resistance in the clinic. Apart from exerting impressive effects on overcoming acquired resistance to osimertinib, MEK/ERK inhibition is also very effective in delaying or abrogating the emergence of osimertinib acquired resistance in the preclinical setting. Rather than waiting for the development of acquired resistance to osimertinib, we can take action early to stop or delay this undesired process to maximize the benefit of the treatment for patients. One potential concern is that the application of the combination of osimertinib and a MEK or ERK inhibitor, which vertically target the same MEK/ERK signaling pathway, for prolonged treatment periods may also increase the risk of toxicity for patients while augmenting the therapeutic efficacy. Since NSCLC cell lines with wild-type EGFR were insensitive to the combination of osimertinib with MEK/ERK inhibition, this combination is not likely to affect the growth of normal cells or tissues that usually have wild-type EGFR. Indeed, even the concurrent combination for over 3 months did not exhibit apparent toxicity in our *in vivo* study, indicating its safety.

Another important finding in this study is the promising effect of MEK/ERK inhibition when given in different intermittent schedules. It is apparent that limited administration of an MEK or ERK inhibitor could achieve similar or even better effects compared with the concurrent treatment in delaying or abrogating the development of acquired resistance to osimertinib. In this way, we can maintain the therapeutic efficacy of the combination while reducing the patient’s burden of drug intake and avoiding potentially increased toxicity. Another advantage for this strategy is that we do not need to make changes to the currently approved treatment protocol of osimertinib when administering a MEK or ERK inhibitor.

In summary, our findings in preclinical models have demonstrated the effectiveness of targeting MEK/ERK signaling for preventing or delaying the emergence of acquired resistance to osimertinib and likely other third-generation EGFR-TKIs. Together with the outcomes from other studies [9], there is a strong rationale in support of clinical studies to validate the strategy of targeting the MEK/ERK pathway to delay or prevent the emergence of acquired resistance to osimertinib or other third-generation EGFR-TKIs. An early and active action that interferes with the process of developing acquired drug-resistance may substantially improve the outcomes of EGFR-mutant NSCLC patients treated with osimertinib or other third-generation EGFR-TKIs.
